# Simultaneous organic/inorganic phosphate solubilization by *Staphylococcus succinus* NG-9 under saline-alkali conditions: Insights into its characteristics, mechanisms, and potential applications

**DOI:** 10.1128/spectrum.00490-25

**Published:** 2025-07-31

**Authors:** Xue Xie, Zhongshun Xu, Longzhan Gan, Chunbo Dong, Ting Zhang, Ya Huang, Tengxia He, Yongqiang Tian, Xiao Zou

**Affiliations:** 1 Institute of Fungus Resources, Guizhou Key Laboratory of Agricultural Microbiology/Key Laboratory of Plant Resource Conservation and Germplasm Innovation in Mountainous Region (Ministry of Education), College of Life Sciences, Guizhou University 71206, Guiyang, Guizhou Province, China; 2 Key Laboratory of Leather Chemistry and Engineering (Ministry of Education), College of Biomass Science and Engineering, Sichuan University 12530, Chengdu, Sichuan Province, China; University of the Philippines Los Baños, Laguna, Philippines

**Keywords:** phosphate-solubilizing bacteria, phosphate solubilization mechanism, saline-alkali stress, genomic analysis, metabolites, biofertilizers

## Abstract

The high salinity and alkalinity of saline-alkali soil limit the growth of plants and agricultural productivity worldwide, and plant growth-promoting rhizobacteria can effectively improve the tolerance of crops to saline-alkali stress. The highly efficient bacterial strain NG-9 that can solubilize phosphate was isolated from a rhizosphere soil sample in saline-alkali land and identified as *Staphylococcus succinus* based on analyses of its phenotype and 16S rRNA sequence. It showed a remarkable ability to solubilize both inorganic phosphate (up to 450.36 mg/L) and organic phosphate (up to 333.15 mg/L) under saline-alkali conditions. In addition, this strain had numerous plant growth-promoting traits at 5.0% NaCl and pH 9.0, including but not limited to the production of 1-aminocyclopropane-1-carboxylate deaminase (7.37 µmol α-ketobutyric acid/mg protein/h), siderophores (36.63%), indole-3-acetic acid (4.14 µg/mL), and extracellular polymeric substances (1.04 g/L). Furthermore, genomic and metabolic analyses confirmed the presence of multiple functional genes and key metabolites related to the promotion of plant growth and ability to adapt to saline-alkali conditions. Notably, the germination rate, length and numbers of roots, and shoot length of wheat (*Triticum aestivum*) seeds increased by 1.04%–162.5%, 13.18%–116.32%, 20.22%–42.86%, and 20.78%–124.42%, respectively, when *Staphylococcus succinus* NG-9 was inoculated under saline-alkali conditions. These findings strongly suggest that *S. succinus* NG-9 can be developed as a multifaceted biofertilizer to reclaim and utilize saline-alkali land.

**IMPORTANCE:**

Plant growth-promoting rhizobacteria can effectively improve the tolerance of crops to saline-alkali stress. A highly efficient phosphate-solubilizing bacterial strain was isolated from a rhizosphere soil sample in saline-alkali land and identified as *Staphylococcus succinus* NG-9. This strain showed a remarkable ability to solubilize both inorganic phosphate and organic phosphate under saline-alkali conditions. In addition, this strain was subjected to an in-depth study of the mechanisms and potential applications using genomic sequencing and annotation, untargeted metabolomics, and a seed germination test. These results strongly suggest that *S. succinus* NG-9 can be developed as a multifaceted biofertilizer to reclaim saline-alkali lands.

## INTRODUCTION

As one of the major abiotic environmental stressors, soil salinization can adversely affect the growth and yield of crop plants, thereby negatively influencing global food security (1). Owing to the climatic and anthropogenic factors, excessive concentrations of soluble salts often occur in the soil. This has severe effects on the quality and health of soil by changing its physicochemical properties and biological communities (2). These phytotoxic effects manifest through osmotic imbalance, ion toxicity, and nutrient deficiency and induce metabolic dysregulation that can culminate in the death of plants at elevated levels of salinity (3). Despite sustained efforts to mitigate these stressors, the global amount of saline-affected soils continues to expand (4).

Plants have developed sophisticated defense mechanisms to manage saline-alkali stress, thus maintaining their growth, development, and cellular processes (5). As a perennial halophyte, the grass *Neotrinia splendens* is remarkably adaptable to its environment and has evolved robust resistance to both salt and drought stress through long-term ecological adaptation (6). Its extensive root system significantly contributes to the fixation of sand and conservation of water in saline areas (7). In addition, this constructive species can provide a major forage resource that is highly palatable and nutritious for local livestock in the arid alkaline regions, and its stalks have long been used as a superior raw material to make paper (8).

The group of bacteria known as plant growth-promoting rhizobacteria (PGPR) has an essential role in increasing soil fertility, enhancing plant growth, and reducing the extensive use of chemical fertilizer over previous decades (9). These root-colonizing bacteria secrete regulatory compounds, including phytohormones that promote the development of plants and improve the acquisition of nutrients through the solubilization and mineralization of organic matter (10). Notably, halotolerant PGPR (HT-PGPR), a specialized subgroup that thrives at a concentration of 1%–25% NaCl (11), demonstrates remarkable effects that enhance the growth of plants under saline conditions. The pretreatment of seeds with strains of HT-PGPR significantly enhances their germination and seedling growth in highly saline environments (12). Under these conditions, HT-PGPR not only retain the ability to promote plant growth, such as the biosynthesis of extracellular polymeric substances (EPS), increased solubility of nutrients, and the production of siderophores, but also mitigate the detrimental effects of salt on plants by different molecular and physiological mechanisms (13). These include the modification of root systems by the production of indole-3-acetic acid (IAA), scavenging of reactive oxygen species (ROS) by the production of a variety of antioxidant molecules, and osmotic protection by the synthesis of osmolytes (14). Furthermore, HT-PGPR can limit plant diseases through several antagonistic mechanisms against phytopathogens or by the induction of systemic resistance in plants (15).

Saline-alkali soils with elevated pH values and contents of Na+ significantly impair the bioavailability of phosphorus, thus limiting its uptake by the plant and consequently affecting its growth (16). As a vital nutrient, phosphorus is crucial for the biosynthesis of nucleic acids, membrane lipid metabolism, photosynthesis, and root development. Phosphorus-solubilizing microorganisms (PSMs) enhance the availability of phosphorus through the following two key mechanisms: (i) the solubilization of inorganic phosphate by secreting organic acids, such as gluconic and citric acids, and (ii) the mineralization of organic phosphate using phosphatase and phytases (17). Notably, some bacterial strains possess both capabilities (18). Nevertheless, only several phosphate-solubilizing microorganisms that can solubilize both organic and mineral sources of soil phosphorus have been identified to date. Since the beginning of the last century, many PSMs have been isolated, such as species of *Bacillus* (19), *Acinetobacter* (20), *Erwinia*, *Raoultella* (21)*, Pantoea* (22), *Flavobacterium* (23), *Enterobacter* (24), and *Staphylococcus* (25). However, most of the known PSMs are unsuitable for saline-alkali agriculture owing to their sensitivity to salt. This highlights the critical need to isolate halotolerant PSMs that can both solubilize and mineralize phosphate for the sustainable agricultural uses of saline soil.

A deep genomic/untargeted metabolomics analysis of the HT-PGPR that are involved in improving crop productivity, particularly halotolerant PSMs, will help develop successful agricultural practices that benefit from the use of these types of PSMs. In this study, we evaluated the phosphate solubilization and plant growth-promoting activity of phosphate-solubilizing microorganisms isolated as NG-9 from the rhizosphere soil of *Neotrinia splendens*. We optimized the media and culture conditions to improve the efficiency of the strains to solubilize phosphate and studied the mechanisms of their phosphate solubilization and promotion of the growth of plants, including insights into their genomics and untargeted metabolomics. Finally, we analyzed the influence of *Staphylococcus succinus* NG-9 on the germination of seeds in greenhouse experiments.

## MATERIALS AND METHODS

### Isolation of the phosphate-solubilizing bacterial strains

Rhizosphere soil was sampled from the root zone of *Neotrinia splendens* that grows in saline-alkali land in Haidong, Qinghai Province, China. The bacteria were isolated on modified National Botanical Research Institute’s phosphate growth (NBRIP) agar medium that contained the following: 10 g/L glucose, 5 g/L Ca_3_(PO_4_)_2_, 0.12 g/L MgSO_4_, 0.2 g/L KCl, 0.1 g/L (NH_4_)_2_SO_4_, and 18 g/L agar, supplemented with 7% NaCl (wt/vol) and adjusted to pH 9.0, using standard serial dilution and plating techniques (26). After incubation at 30°C for 5–7 days, single colonies with a clear halo zone were considered to be phosphate solubilizers and then selected for further purification and validation. Based on its degree of phosphate solubility, the strain designated NG-9 was subjected to an additional study of stress tolerance and plant growth promotion. The potential of the bacterial strains to solubilize phosphate was preliminary estimated using the solubilization index, which was calculated as follows:


$$Solubilization\;Index\;(SI)=\frac{Colony\;diameter+Halozone\;diameter}{Colony\;diameter}$$

### Identification of the selected isolate

#### Phenotypic characterization

The morphological characteristics of strain NG-9 were examined by light microscopy (Leica ICC50 W, Wetzlar, Germany) and scanning electron microscopy using cells at the exponential growth phase as described in Bergey’s Manual of Determinative Bacteriology (27). The colony characteristics were observed after 72 h of routine cultivation on Luria–Bertani (LB) agar that contained 7% NaCl (wt/vol) at 30°C. The activities of oxidase and catalase were tested using 1% tetramethyl-*p*-phenylenediamine (wt/vol) and 3% hydrogen peroxide (vol/vol), respectively. The production of indole and the urease and Voges–Proskauer tests were conducted as described by Mata et al. (28).

#### Analysis of the 16S rRNA sequence

A DNA template of strain NG-9 was prepared using the Chelex-100 boiling method, and its 16S rRNA gene fragments were amplified by PCR in a T100 Thermal Cycler (Bio-Rad, Hercules, CA, USA) with the universal bacterial primers 27F (5′-AGAGTTTGATCCTGGCTCAG-3′) and 1492R (5′-GGTTACCTTGTTACGACTT-3′) as previously described (29). The PCR products were purified using a commercial DNA purification kit, inserted into a pUCm-T cloning vector, and then sequenced by Shanghai Sangon (Shanghai, China). The sequence obtained was compared with the reference sequences available from the EzBioCloud server (https://www.ezbiocloud.net/) to identify its nearest relatives and calculate pairwise sequence similarities. A neighbor-joining phylogenetic tree based on the aligned 16S rRNA gene sequences was reconstructed with MEGA 11 using Kimura’s two-parameter model (30).

### Optimization of the cultural conditions for phosphate solubilization

The effects of key environmental factors on the ability of strain NG-9 to solubilize phosphate were evaluated by a series of experiments. First, the effects of salinity were tested by culturing the strain in modified NBRIP broth with varying concentrations of NaCl (initial pH 8.0) at 30°C and 200 rpm for 7 days. The optic density(OD) values determined the optimal salinity. Next, the pH was optimized in media at different pH values, each containing 5% NaCl. The amount of phosphate solubilization after incubation was determined by spectrophotometry. Each assessment of the carbon (C)/nitrogen (N) sources, glucose and (NH_4_)_2_SO_4_, was conducted by replacing the elements with alternative sources, including fructose, lactose, sucrose, maltose, starch, mannitol, KNO_3_, NaNO_3_, or NH_4_Cl, in pH 9.0 media, with subsequent measurements of phosphate solubilization. Finally, the effects of temperature were examined across 20°C–40°C in optimized media (5% NaCl, pH 9.0). The pH values and degree of phosphate solubilization were used to identify the ideal growth temperature. Each optimized parameter was systematically applied to subsequent experimental phases to establish comprehensive cultivation conditions for strain NG-9.

### Quantitative determination of the ability to solubilize phosphate

The ability of strain NG-9 to solubilize phosphate was quantified by monitoring the amount of available phosphorus in modified NBRIP broth supplemented with tricalcium phosphate or calcium phytate as the substrate (31). After cultivation under different operating conditions, the fermented broth was centrifuged at 7,000 × *g* for 5 min, and the supernatant was used as the source of a test sample. The content of available phosphorus was measured by the molybdenum-antimony anti-spectrophotometric method at 700 nm using a Multiskan SkyHigh microplate spectrophotometer (Thermo Fisher Scientific, Waltham, MA, USA) (32) where KH_2_PO_4_ was used as the standard.

### Assessment of the multi-functional plant growth-promoting traits

#### 1-Aminocyclopropane-1-carboxylate (ACC) deaminase activity

The activity of ACC deaminase was evaluated by monitoring the amount of α-ketobutyric acid generated from the cleavage of ACC by strain NG-9 (33). Briefly, the strain was incubated in Dworkin and Foster (DF) minimal broth that contained 5% NaCl and 3 mM ACC as the sole source of N at 30°C for 2 days. The total amount of α-ketobutyric acid was then determined using a standard curve at 540 nm. The total protein content was examined by the Bradford method (34). The ACC deaminase activity was calculated as follows:


$$ACC\;deaminase\;activity=\frac{C_{k}}{C_{p}\times \Delta T}$$

where *C_p_*, *C_k_*, and ∆*T* are the total protein concentration (g/mL), total α-ketobutyric acid concentration (mol/L), and the sample reaction time (h), respectively.

#### Siderophore production

The production of siderophores by strain NG-9 was assessed using chrome azurol S (CAS) assays (35). The cultures were analyzed qualitatively by plating on CAS agar followed by incubation at 30°C for 5–7 days. The formation of an orange halo indicated the production of siderophores. A quantitative analysis was conducted by inoculating the strain into modified King’s B broth that contained 5% NaCl (wt/vol) followed by cultivation at 200 rpm at 30°C for 2 days. After centrifugation, the fermentation supernatant was incubated with the CAS reagent (1:1 vol/vol) for 1 h at room temperature, and the absorbance at 630 nm was measured. The production of siderophores was expressed as the percent siderophore unit (PSU) as described by Cruz et al. (36). The PSU was calculated as follows:


$$PSU=\frac{A_{b}-A_{s}}{A_{b}}\times 100\text{\textbackslash{}\% }$$

where *A_b_* and *A_s_* are the absorbances of the blank and the sample, respectively.

#### Production of indole-3-acetic acid

The production of IAA was quantified as described by Bunsangiam et al. (37). Strain NG-9 was cultured in LB broth with 0.1% tryptophan and 5% NaCl (wt/vol) at 30°C and 200 rpm for 7 days. After centrifugation, the Salkowski reagent, which contained 300  mL of 95% sulfuric acid, 15 mL of 0.5 M ferric chloride, and 500  mL of H_2_O, was added to the supernatant at a ratio of 4:1 and incubated in the dark for 15 min. The absorbance at 530 nm was measured on a microplate reader using a 0 to 500 mg/L standard curve of IAA.

#### Production of exopolysaccharides

The strain was first grown in a nutrient broth that contained 10 g/L tryptone, 5 g/L yeast extract, 50 g/L NaCl, 20 g/L sucrose, 0.5 g/L NaNO_3_, 0.5 g/L MgSO_4_, and 0.5 g/L K_2_HPO_4_ at pH 9.0 ± 0.2 at 30°C for 2 days to quantitatively analyze the exopolysaccharides (EPSs). The crude EPSs were extracted from the fermented broth as previously described and then quantified by the phenol–sulfuric acid method at 490 nm (38).

#### Biofilm formation

The biofilm was quantified in 24-well microtiter plates as described by Wang et al. (39). First, the strain was cultivated in 24-well plates that contained LB broth with different concentrations of NaCl and 2% inoculum (vol/vol). After incubation at 30°C for 2 days, each well was emptied and then washed three times with phosphate-buffered saline. The samples were then fixed with methanol for 20  min and dried under natural conditions. The biofilms were stained with 200  µL of 0.1% crystal violet (CV) for 10  min and then washed clear with water. The CV dye was solubilized in 500  µL of 33% acetic acid, and its intensity was measured at 590  nm.

### Genomic sequencing and annotation

The total DNA of strain NG-9 used for genome sequencing was extracted using a Tissue Genome DNA Extraction kit (Majorbio Biotech, Shanghai, China) according to the manufacturer’s instructions. The genome was sequenced by Majorbio Bio-Pharm Technology Co., Ltd. (Shanghai, China) using the PacBio Sequel and Illumina NovaSeq PE150 platforms. The data integrity was maintained by filtering the low-quality reads with fastp v.0.23.0, which resulted in high-quality contigs. The genes were annotated and their functions predicted using a combination of GeneMarkS, Pfam, Swiss-Prot, Non-Redundant Protein (NR), Gene Ontology (GO), Kyoto Encyclopedia of Genes and Genomes (KEGG), and Clusters of Orthologous Groups (COG) databases. The data were visualized using the online platform Majorbio Cloud Platform (https://cloud.majorbio.com).

### Metabolomic analysis

The fermented NBRIP broth (5% NaCl, initial pH 9.0 and 30°C) of strain NG-9 was sampled at 0, 1, and 7 days for an untargeted metabolomic analysis because there were large changes in the pH and dissolved phosphorus. Each sample was processed as described by Yu et al. (40). The metabolites for each sample were then detected using a Thermo UHPLC-Q Exactive HF-X (Thermo Fisher Scientific) and identified using Progenesis QI v.2.3 software (41). The metabolites that were identified were annotated by the KEGG database and Human Metabolome Database (HMDB). A principal component analysis (PCA) and partial least squares discriminant analysis (PLS-DA) were conducted using ropls (R packages) v.1.6.2 and scipy (Python) v.1.0.0, respectively, and the variable importance in the projection (VIP) value of each metabolite was obtained. The VIP value refers to the variable-projected importance of the first principal component of the PLS-DA model, which indicates the contribution of metabolites to the grouping.

### Measurement of the activities of phosphatase and phytase

The phosphatase activities were measured as described by Landa-Acuña et al. (42) and Suleimanova et al. (22) with modifications. Strain NG-9 was cultured in NBRIP broth (5% NaCl, pH 9.0) with tricalcium phosphate or calcium phytate as the source of phosphorus. Cell-free supernatants from 30°C/200 rpm cultures were assayed. One unit (U) represented 1 µg *p*-nitrophenol released from *p*-nitrophenyl phosphate per minute per milliliter at pH 6.5 (acid phosphatase) or 11.0 (alkaline phosphatase). The phytase activity was determined via sodium phytate degradation using the molybdenum vanadium method (43) under identical culture conditions. One unit of phytase = 1 µmol available phosphorus released per minute per milliliter from the fermentation broth.

### Seed germination test

Unprimed wheat (*Triticum aestivum*) seeds from the local market were selected for a seed germination assay as previously described with minor modifications (44). The seeds were surface sterilized with 75% ethanol for 5 min and then treated with 1% NaClO for 5 min followed by three rinses with sterile water. Next, the seeds were soaked in the bacterial suspension for 2 h and then in sterile water. They were then placed on filter paper in Petri dishes. The control utilized sterile water instead of the bacteria. The filter papers for each set were moistened with 0, 50, 100, 150, and 200 mM NaCl at a consistent pH of 9.0. All the seeds were then incubated in a 25°C incubator (12/12 h light/dark, relative humidity 75%) for 14 days. The seed germination and seedling growth were observed and recorded regularly from day 1 to day 14 as previously described (45).

The germination index (GI) was calculated as follows:


$$GI=\frac{No.\;of\;germinated\;seed}{days\;of\;the\;first\;count}+\frac{no.\;germinated\;seed}{days\;of\;the\;final\;count}\times 100$$

The relative germination (RG) and vigor index (VI) were calculated as follows:


$$RG=\frac{number\;of\;gemination\;in\;each\;treatments}{number\;of\;gemination\;in\;control}\times 100$$

VI={(mean root length+mean shoot length)}×% of germination

### Statistical analysis

The data were processed and the graphs plotted using SPSS 24.0 (IBM, Inc., Armonk, NY, USA) and Origin 2021 (OriginLab, Northampton, MA, USA), respectively. The data were expressed as the mean ± SD, and the differences among the groups were determined by a Duncan’s multiple range test. *P* < 0.05 was considered to be significant.

## RESULTS

### Screening of the phosphate-solubilizing isolates under saline-alkali stress

**Fig 1:**
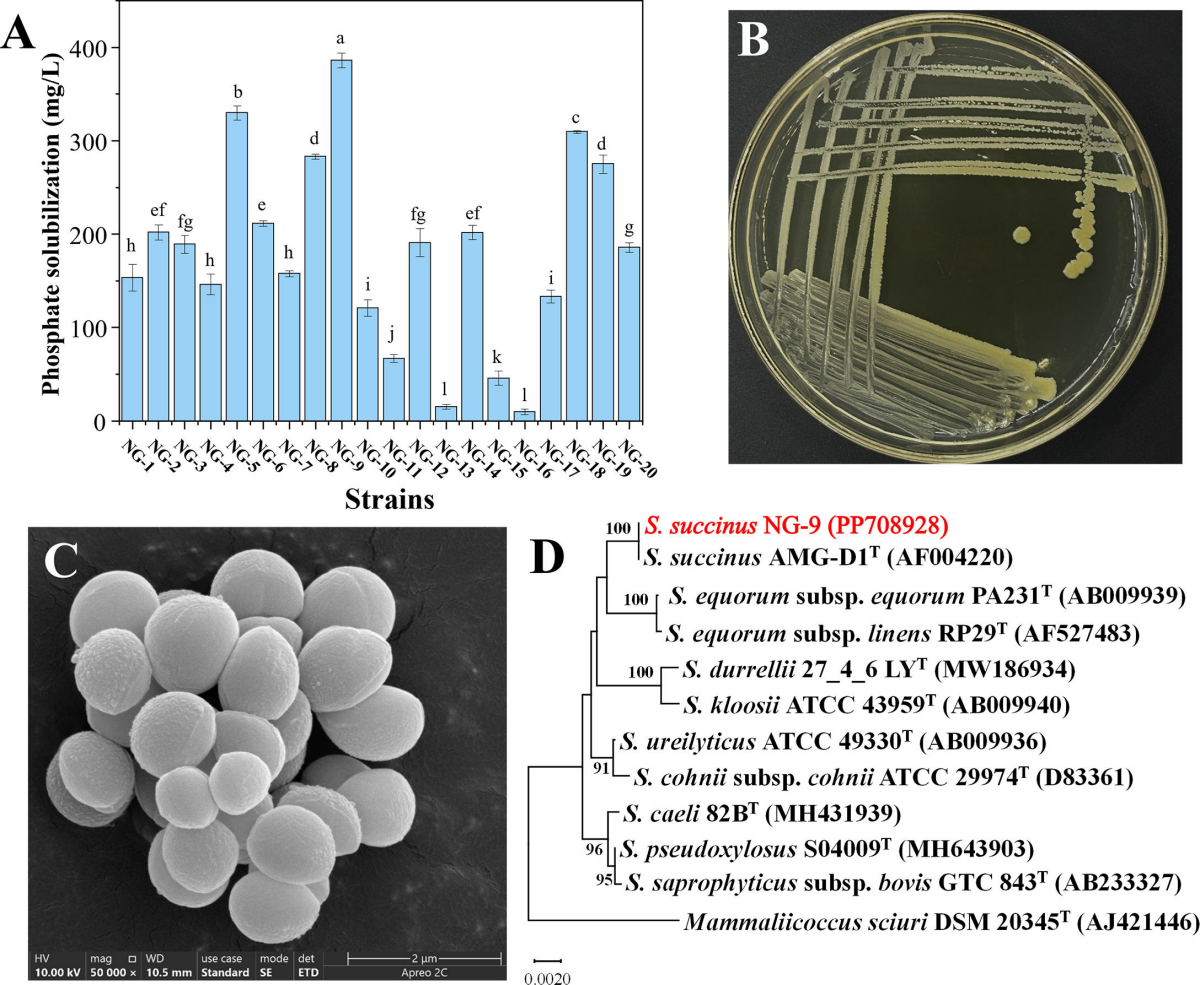
Selection, morphological characterization, and phylogenetic analysis of strain NG-9. Inorganic phosphate-solubilizing ability for the new isolates (**A**); colony characteristics on LB agar plates (**B**); cell morphology by SEM (**C**); neighbor-joining tree based on 16S rRNA gene sequences (**D**). Different lowercase letters indicate the significant differences (*P* < 0.05).

A total of 20 bacterial strains that could solubilize phosphate were isolated from the soil samples collected from the root zone of *Neotrinia splendens* using modified NBRIP agar medium. These strains grow on NBRIP supplemented with 7.0% NaCl and pH 8.0 and solubilize phosphates. The quantitative determination of their ability to solubilize phosphate under saline-alkali conditions showed that the contents of soluble phosphorus in the fermentation broth of these 20 isolates ranged from 9.63 ± 2.91 to 386.13 ± 7.78 mg/L (Fig. 1A). The content of soluble phosphorus in the fermentation broth of strain NG-9 was approximately 386.13 mg/L, which was the highest among the isolates in the same batch. This value is higher than those previously reported and was therefore selected for further analysis.

### Morphological and molecular identification of strain NG-9

Strain NG-9 was routinely cultured on LB agar that contained 7.0% NaCl at pH 8.0 for 72 h, and the colony was yellow with irregular edges and a dry surface. It was 1.5 mm–3 mm in diameter (Fig. 1B). The strain was gram-positive, and its cells were spherical under a light microscope. The structure of strain NG-9 under a scanning electron microscope is shown in Fig. 1C. The diameter of the bacterium was 0.7 µm–1.3 µm. The bacterial culture reacted positively to urease and produced indole, while it was negative for oxidase and catalase and the Voges–Proskauer test (Table S1). The results were comparable to those of Lambert et al. (46) on *Staphylococcus succinus*.

The 16S rRNA gene of strain NG-9 was sequenced using the EzBioCloud database, which revealed 100.0% similarity with *Staphylococcus succinus* AMG-D1^T^. MEGA 11 was used to conduct a phylogenetic analysis with the neighbor-joining method, and 1,000 bootstrap replicates showed the closest evolutionary relationship between strain NG-9 and *S. succinus* AMG-D1^T^ (Fig. 1D). They formed a distinct clade with strong bootstrap support (100%). Based on these molecular phylogenetic results, along with comprehensive physiological and biochemical characterization, the high-performance phosphate-solubilizing strain was taxonomically identified as *Staphylococcus succinus* NG-9 and deposited in the NCBI GenBank database under accession number PP708928.

### Optimization of conditions for the phosphate solubilization activity

#### Salinity

**Fig 2:**
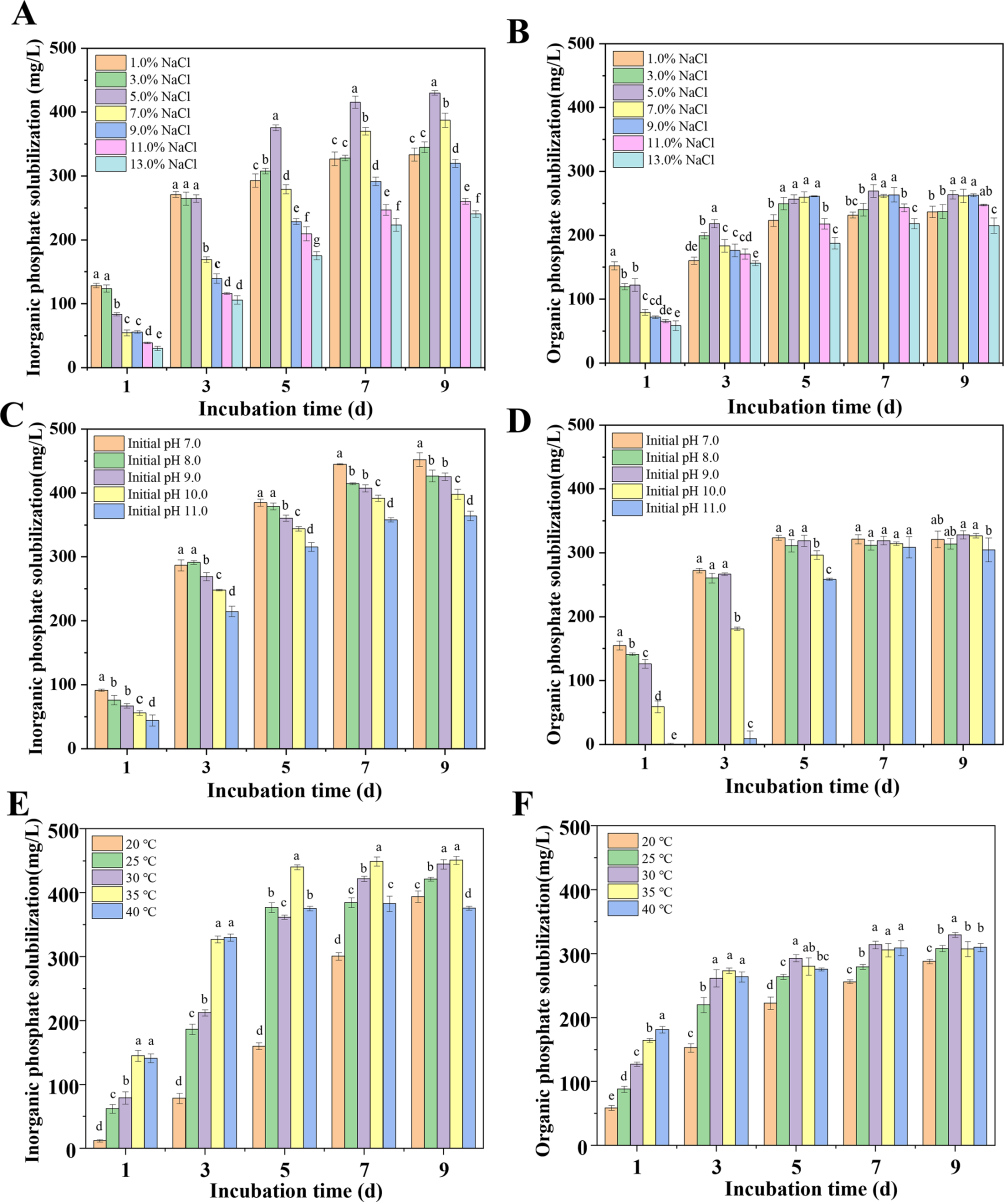
Effects of typical environmental factors on inorganic/organic phosphate solubilization by *S. succinus* NG-9. NaCl concentrations (**A and B**); initial pH values (**C and D**); culture temperatures (**E and F**). Different lowercase letters indicate significant differences (*P* < 0.05).

The effects of different concentrations of NaCl on the dissolution of inorganic and organic phosphates in *S. succinus* NG-9 are shown in Fig. 2 and S1. This study showed that NG-9 grew at1.0%–13.0% NaCl and effectively dissolves phosphate. On day 9 of culture, the solubility of inorganic phosphate ranged from 240.50 ± 5.02 mg/L to 430.07 ± 3.66 mg/L, while that of the organic phosphate ranged from 215.34 ± 11.98 mg/L to 263.54 ± 6.59 mg/L. These results indicate that the microorganism is tolerant to salt. A sharp decrease in culture pH from 8.0 to approximately 5.5 was observed on the 1st day of fermentation, followed by a slight decrease. However, compared with the pH value, the dissolution of phosphate by *S. succinus* NG-9 increased stably after 1 day and reached its maximum level of dissolution at 7 days of incubation. In the relatively low salinity range, the levels of salinity positively affected the ability of bacterial isolates to solubilize phosphate. It was apparent that the highest amount of phosphate dissolved (415.44 ± 9.28 mg/L and 269.20 ± 9.92 mg/L for the inorganic and organic phosphates, respectively) was achieved with 5.0% NaCl, while the lowest value of phosphate dissolved (240.50 ± 5.02 mg/L and 215.34 ± 11.98 mg/L for the inorganic and organic phosphates, respectively) was obtained in a medium that contained 13.0% NaCl. These results demonstrate that 5.0% NaCl is the optimal concentration to observe the dissolution of both inorganic and organic phosphate dissolution.

#### pH

The effect of the initial pH of the medium on the dissolution of inorganic and organic phosphates and the pH was studied for this isolate. As shown in Fig. 2 and S1, different initial pH values had varying effects on the ability of *S. succinus* NG-9 to solubilize phosphate, and this ability decreased as the pH increased. Notably, no dissolution of the organic phosphates was observed at pH 11.0 on day 1. The phosphate-solubilizing ability increased over time and reached its maximum on day 7 when the amount of dissolved inorganic phosphate ranged from 357.74 ± 3.25 mg/L to 444.94 ± 0.84 mg/L, while the dissolved organophosphate ranged from 32.36 ± 7.17 mg/L to 308.69 ± 16.36 mg/L. Although the highest level of phosphate dissolution occurred at pH 7.0, pH 9.0 was selected as the initial pH of the NBRIP medium to optimize the solubilization of phosphate since the strain was intended to promote plant growth and improve the soil in saline-alkali soil. The results demonstrated that *S. succinus* NG-9 effectively solubilized phosphate under high alkali stress, indicating its strong tolerance to extreme alkaline conditions.

#### Carbon and nitrogen sources

Glucose in the media was replaced with various C sources, namely mannitol, soluble starch, and fructose, to obtain the optimal phosphate-solubilizing ability. As shown in Fig. S2A and B, glucose was the best C source among those tested for their ability to solubilize organic phosphates in media containing both types of phosphate. However, the phosphate was nearly undetectable in the inorganic medium when mannitol served as the C source. The optimal ability to solubilize phosphates was observed in the glucose media and was 413.37 ± 4.82 mg/L and 281.81 ± 3.86 mg/L for inorganic and organic phosphates, respectively. The replacement of glucose with mannitol, soluble starch, and fructose resulted in a lower rate of phosphate solubilization. Moreover, the maximum ability to solubilize phosphate was observed (432.78 ± 7.39 mg/L and 333.15 ± 9.41 mg/L for inorganic and organic phosphates, respectively) when ammonium chloride (NH_4_Cl) was used as the N source (Fig. S2C and D). Therefore, we found that glucose and NH_4_Cl were the best C and N sources for *S. succinus* NG-9 to dissolve phosphate, respectively.

#### Temperature

Temperature plays a vital role in the growth and physiological activity of PSMs. The effect of incubation temperature on the ability to solubilize phosphate was studied for the isolate. As shown in Fig. 2 and S1, the ability of *S. succinus* NG-9 to solubilize phosphate increased with the increase in the temperature of the culture. The content of soluble phosphates and decrease in pH in the broth at different times varied depending on the temperature. In general, the phosphate solubility was maximal or close to maximal (444.26 ± 7.26 mg/L and 329.10 ± 3.95 mg/L for inorganic and organic phosphates, respectively) at 30°C. It was notable that the bacteria were less effective at dissolving phosphate at 20°C and 25°C during the early fermentation stage, and the decrease in pH was relatively gradual, possibly because the low temperature could have affected the growth and metabolism of the bacteria.

### Plant growth-promoting traits under saline-alkali stress conditions

Strain *S. succinus* NG-9 exhibited multiple plant growth-promoting traits under saline-alkaline conditions (5.0% NaCl, pH 9.0) as summarized in Table S2. The ACC deaminase activity of strain NG-9 was quantified in DF minimal medium using ACC as the sole N source. The vigorous growth of this strain under these conditions confirmed its capacity for ACC deaminase activity, which was measured at 7.37 ± 0.40 µmol α-ketobutyric acid/mg protein/h. The strain actively produced siderophores since it generated 36.63 ± 0.06% siderophore units after 48 h of cultivation as quantified by the CAS assay. NG-9 was also shown to biosynthesize phytohormones since the isolate produced 4.14 ± 0.69 µg/mL IAA when cultured in LB medium supplemented with L-tryptophan for 7 days as measured spectrophotometrically at 530 nm. The yield of EPS reached 1.04 ± 0.33 g/L under identical saline-alkaline conditions as determined by the phenol-sulfuric acid method at 490 nm. The ability to form biofilm was systematically evaluated across a gradient of NaCl concentrations (1.0%–13.0%) as shown in Fig. S3. The absorbance values at 590 nm revealed the following characteristic pattern that was dependent on the salinity: the production of biofilm increased progressively at lower concentrations of NaCl (1.0%, 3.0%, 5.0%, and 7.0%) and reached its maximal yield at 7.0% NaCl (0.76 ± 0.06), followed by gradual attenuation at higher salinities.

### Genomic analysis of *S. succinus* NG-9

#### General genome features

**Fig 3:**
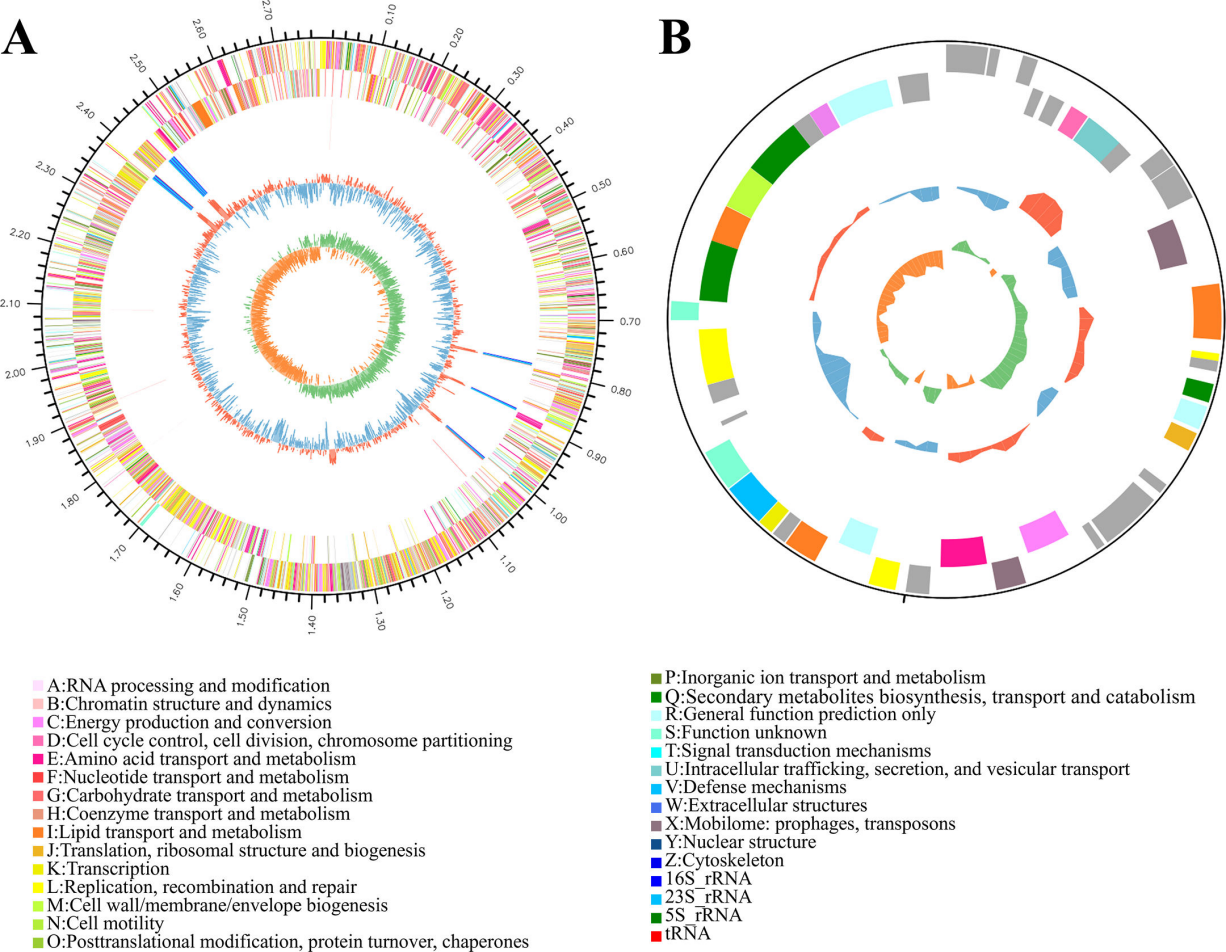
Circular genome maps of the *S. succinus* NG-9 chromosome (**A**) and plasmid (**B**).

The genome of *Staphylococcus succinus* NG-9 was sequenced and analyzed. Hybrid assembly revealed one circular chromosome (2,774,080 bp; GC content, 33.14%) and one plasmid that was designated pC2014-2 (38,187 bp; GC content, 30.81%) (Fig. 3). The complete sequence of the strain *Staphylococcus succinus* NG-9 was submitted to GenBank with accession number CP195802 and CP195803. A total of 2,646 genes were identified, including 55 tRNA and 25 rRNA genes, in which 2,632, 2,035, 2,251, 2,201, and 1,848 genes were annotated by the NR, KEGG, COG, Swiss-Prot, and GO databases, respectively. Furthermore, 2,251 genes were classified into 23 COG categories. Most were associated with functions, such as carbohydrate transport and metabolism, amino acid transport and metabolism, general function prediction only, translation, ribosomal structure and biogenesis, transcription, coenzyme transport, and metabolism.

#### Genes related to the promotion of plant growth and stress responses

The genome annotation of *S. succinus* NG-9 revealed functional genes linked to phosphate solubilization, plant growth promotion, and salt stress tolerance (Table 1). The bacteria solubilized inorganic phosphate via multiple pathways, including the production of organic/inorganic acids, direct oxidation, and siderophore biosynthesis, which suggests a complex metabolic network. Key phosphate metabolism genes were identified, including *eno* (enolase, a phosphopyruvate hydratase), *ppaC* (pyrophosphatase), and *ppx-gppA* (exopolyphosphatase); the latter is crucial for the conversion of polyphosphate to phosphate (47). Pst is an ATP-binding cassette transporter (ABC transporter) that is driven by ATP and has a high affinity to take up phosphate. The Pst system is generally composed of the phosphate ABC transporter substrate-binding protein PstS, phosphate ABC transporter permease PstC, phosphate ABC transporter permease PstA, phosphate ABC transporter ATP-binding protein PstB, and phosphate signaling complex protein PhoU (48). PstS (*pstS*), PstC (*pstC*), PstA (*pstA*), and PstB (*pstB*) along with PhoU (*phoU*) are all components of the Pst system and have been identified in the genome of *S. succinus* NG-9.

**TABLE 1:** Annotated genes involved in plant growth-promoting traits of *S. succinus* NG-9 under saline-alkali conditions^*a*^

Plant growth-promoting trait	Gene name	Start	End	Gene description
Nitrogen fixation	*gltD*	2457571	2456108	Glutamate synthase subunit beta
	*gltB*	2462091	2457592	Glutamate synthase large subunit
IAA production	*trpB*	1520170	1518956	Tryptophan synthase subunit beta
	*trpC*	1521581	1520799	Indole-3-glycerol phosphate synthase TrpC
	*trpF*	1520802	1520170	Phosphoribosylanthranilate isomerase
	*trpD*	1522581	1521565	Anthranilate phosphoribosyltransferase
	*trpG*	1523146	1522550	Aminodeoxychorismate/anthranilate synthase component II
	*ipdC*	239302	240945	α-Keto acid decarboxylase family protein
Phytase activity	*–*	1151177	1150005	Esterase-like activity of phytase family protein
Siderophore production	*fhuB*	2226693	2225692	Iron ABC transporter permease
	*fhuD*	513090	514001	ABC transporter substrate-binding protein
EPS production	*gumF*	1896933	1897937	Acyltransferase family protein
	*exoZ*	1959034	1957205	Acyltransferase family protein
	*cysE*	1959623	1959315	Metal-sulfur cluster assembly factor
	*wecA*	2126034	2124874	MraY family glycosyltransferase
Biofilm formation	*trpG*	1523146	1522550	Aminodeoxychorismate/anthranilate synthase component II
	*trpE*	1524549	1523143	Anthranilate synthase component I
	*luxS*	826687	826217	S-ribosylhomocysteine lyase
	*wecB*	848759	849889	UDP-N-acetylglucosamine 2-epimerase (non-hydrolyzing)
	*crr*	1465378	1465878	PTS glucose transporter subunit IIA
	*hfq*	1620425	1620213	RNA chaperone Hfq
	*cysE*	1959623	1959315	Metal-sulfur cluster assembly factor
Phosphate solubilization	*eno*	2084807	2083503	Surface-displayed α-enolase
	*gntZ*	1392624	1394033	NADP-dependent phosphogluconate dehydrogenase
	*mdh*	2187247	2186312	Malate dehydrogenase
	*gltA*	1170503	1171621	Citrate synthase
	*thiE*	864050	864688	Thiamine phosphate synthase
	*mqo*	623126	624607	Malate dehydrogenase (quinone)
	*fumC*	1022330	1023715	Class II fumarate hydratase
	*phoA*	674524	675957	Alkaline phosphatase
	*ppaC*	947883	946957	Manganese-dependent inorganic pyrophosphatase
	*ppx-gppA*	526590	525064	Exopolyphosphatase
	*Ldh*	1088364	1089320	L-lactate dehydrogenase
	*pstA*	1507250	1508161	Phosphate ABC transporter permease PstA
	*pstB*	1508251	1509129	Phosphate ABC transporter ATP-binding protein PstB
	*pstC*	1506318	1507247	Phosphate ABC transporter permease subunit PstC
	*pstS*	1505140	1506111	PstS family phosphate ABC transporter substrate-binding protein
	*phnA*	2678269	2677916	Zinc ribbon domain-containing protein YjdM
	*phnB*	268885	269301	Glyoxalase/bleomycin resistance/extradiol dioxygenase family protein
	*Gdh*	661991	662872	SDR family oxidoreductase
	*–*	450202	451101	Phosphatase PAP2 family protein
	*nrnA*	1157999	1158934	Bifunctional oligoribonuclease/PAP phosphatase NrnA
	*bcrC*	1469333	1469947	Phosphatase PAP2 family protein
Saline-alkali tolerance	*deaD*	873662	875182	DEAD/DEAH box helicase
	*cshB*	1306504	1307844	DEAD/DEAH box helicase
	*comFA*	2122924	2121584	DEAD/DEAH box helicase family protein
	*betA*	290261	291943	Choline dehydrogenase
	*betB*	288698	290191	Betaine-aldehyde dehydrogenase
	*treR*	2452521	2451796	Trehalose operon repressor
	*dnaK*	1285743	1287587	Molecular chaperone DnaK
	*dnaJ*	1287736	1288869	Molecular chaperone DnaJ
	*cspA*	1491766	1491966	Cold shock protein CspA
	*corA*	640151	641101	Magnesium/cobalt transporter CorA
	*copA*	378885	376501	Heavy metal translocating P-type ATPase
	*copZ*	376451	376245	Copper chaperone CopZ
	*znuA*	574198	575748	Zinc ABC transporter substrate-binding lipoprotein AdcA
	*znuB*	1309636	1310508	Metal ABC transporter permease
	*znuC*	1308866	1309639	Metal ABC transporter ATP-binding protein
	*proC*	232997	232800	Pyrroline-5-carboxylate reductase
	*proS*	1677361	1675661	Proline—tRNA ligase
	*betB*	288698	290191	Betaine-aldehyde dehydrogenase
	*betA*	290261	291943	Choline dehydrogenase
	*trkA*	1854671	1854012	TrkA family potassium uptake protein
	*trkH*	2030999	2032303	Potassium transporter TrkG

^*a*^ –, Unnamed gene.

Other genes that are known to contribute to the solubilization of inorganic phosphate include *gdh*, which encodes pyrroloquinoline quinone (PQQ)-dependent membrane-bound glucose dehydrogenase (GDH) that directly oxidizes glucose to produce gluconic acid. The protein is composed of 793 amino acids with a molecular weight of approximately 86 kDa that contains a domain for glucose metabolism designated PQQ-dependent membrane-bound GDH. Additionally, the entire NG-9 genome contains several pertinent genes that encode proteins that can solubilize phosphate. They include alkaline phosphatase (*phoA*) and exopolyphosphatase (*ppx-gppA*). These genes enable *S. succinus* NG-9 to be an excellent solubilizer of phosphate, which can release insoluble phosphate from both organic and inorganic sources.

The *S. succinus* NG-9 genome contains plant growth-promoting genes linked to the suppression of pathogens, solubilization of nutrients, and resistance to stress. These activities have been identified during *in vitro* assays. Key genes encode phytase, siderophores, IAA, biofilm/EPS producers, and ACC deaminase (*rimM*), which reduces the amount of ethylene to aid nodulation (49). A whole-genome study of NG-9 revealed numerous IAA biosynthetic genes. These included *ipdC* that encodes indole-3-pyruvate decarboxylase (50). L-tryptophan is an important amino acid that is required for the normal growth and development of plants and also acts as a precursor for plant growth regulators. We found the genes for tryptophan biosynthesis, including *trpB* for tryptophan synthase subunit beta, *trpC* for bifunctional indole-3-glycerol-phosphate synthase, *trpF* for phosphoribosylanthranilate isomerase, *trpD* for anthranilate phosphoribosyltransferase, and *trpG* for the bifunctional anthranilate synthase glutamate amidotransferase component (51). Furthermore, the genome of this strain contains the gene associated with ferrichrome (a hydroxamate-type siderophore)-iron receptor (*fhuBD*) and pyridoxal phosphate-dependent deaminase and D-cysteine desulfhydrase (*dcyD*) (51, 52).

*S. succinus* NG-9 was isolated from saline-alkali soil and grew well on NBRIP supplemented with 7% salt. Genome mining showed that the strain harbored several genes associated with salt tolerance. For example, the genes *betA* and *betB* involved in glycine-betaine biosynthesis were identified in the NG-9 genome. These two genes are reported to be the most important genes associated with salt tolerance (53). Furthermore, trehalose functions as an osmoprotectant under extreme conditions, such as drought, high salt concentration, and osmotic stress (54). The genome of *S. succinus* NG-9 also contains genes related to ion homeostasis, which consist of *mrp*, *trkH*, and *trkA*. Moreover, the genes of the universal stress protein family in the NG-9 genome, including *uspA* and *uspG*, were observed (55). Proline is a significant osmolyte with a multifunctional role, including the maintenance of cytosolic pH and antioxidant activity, and it functions as a molecular chaperone during osmotic stresses (56). In most bacteria, the biosynthesis of proline implicates the united function of γ-glutamyl kinase and 1-pyrroline-5-carboxylate reductase enzymes, which are encoded by *proB* and *proC*, respectively. The genome of *S. succinus* NG-9 also harbors the genes *dnaK* and *dnaJ* that are related to the quality control of proteins. They are both molecular chaperones that play vital roles in stress responses.

### Key metabolites associated with the dissolution of phosphate and saline-alkali tolerance

**Fig 4:**
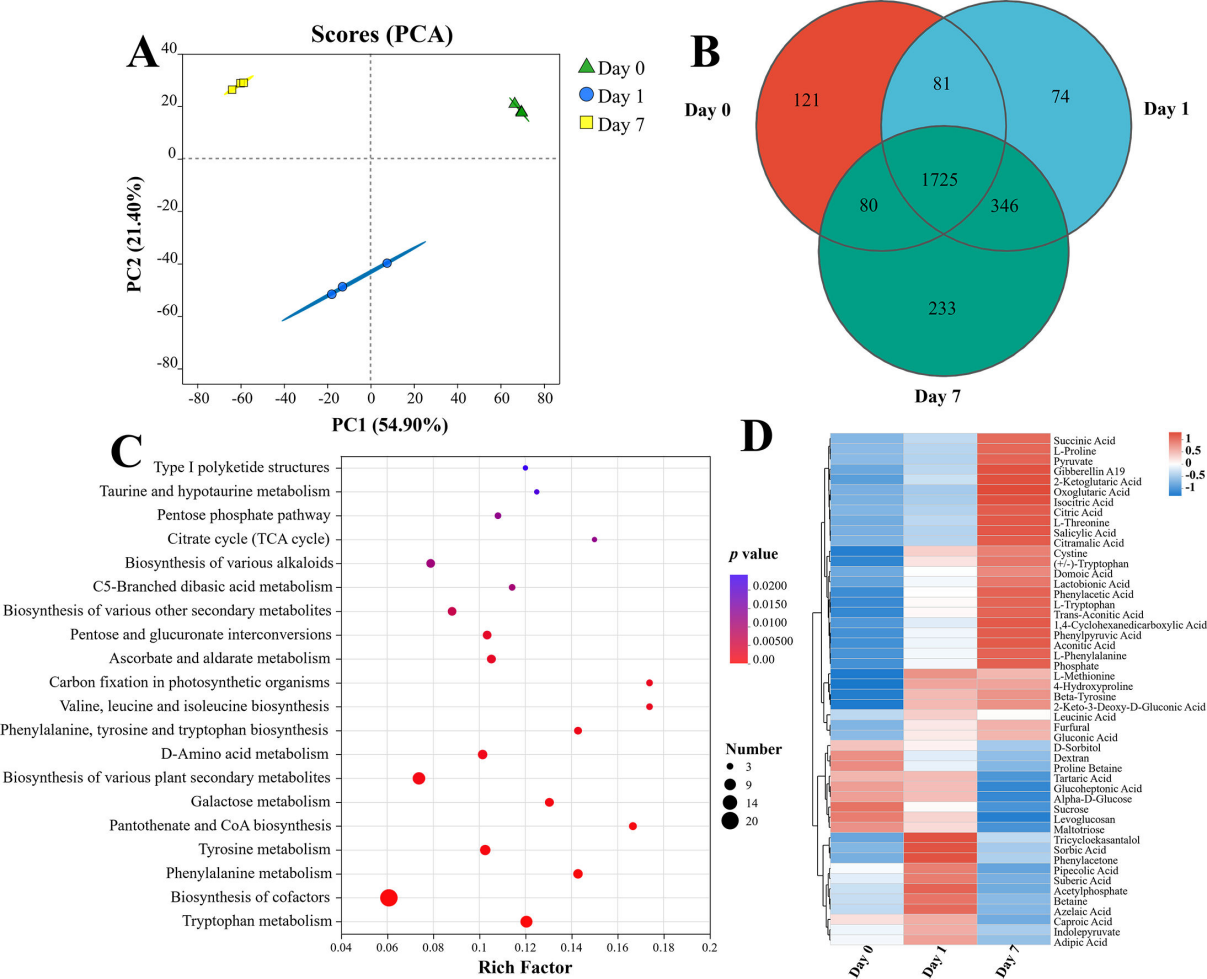
Analyses of differential metabolites in fermented broth of *S. succinus* NG-9 during the incubation period. PCA on days 0, 1, and 7 (**A**); Venn diagram of the number of shared and unique metabolites among the three incubation intervals (**B**); KEGG pathway enrichment analysis for differential metabolites (**C**); clustering heatmap of differential metabolites (**D**).

Untargeted metabolomics was used to study the differentially expressed metabolites in *S. succinus* NG-9 on days 0, 1, and 7. The PCA showed that four samples from each group individually clustered together and were separated from each other (Fig. 4A). These results indicated that the metabolites of days 0, 1, and 7 were significantly different, and there was no significant difference between the replicated samples of each treatment. Therefore, these data were reliable. In addition, a total of 2,007 metabolites were detected on day 0, 2,226 on day 1, and 2,384 on day 7 (Fig. 4B). Among them, a KEGG functional pathway analysis revealed that the functional pathways of these metabolites included tryptophan metabolism, phenylalanine metabolism, the citric acid cycle (TCA cycle), pentose phosphate pathway, and pentose and glucuronate interconversions (Fig. 4C). This finding indicated that these metabolites play important roles in the promotion of plant growth and resistance to saline stress. Previous studies have shown that succinic acid is produced by the TCA cycle; gluconic acid is produced through the pentose phosphate pathway, and the pentose phosphate pathway is also closely related to the C fixation pathway. This elevates the pentose phosphate pathway and could possibly cause it to immobilize more carbon dioxide to improve salt tolerance (57).

A hierarchical clustering analysis of the differential metabolites revealed distinct metabolic patterns across the samples, thus visually highlighting the upregulated/downregulated changes over time (Fig. 4D). The acidolysis primarily involves organic acids secreted by PSMs, such as malic, succinic, and citric acids, which chelate Ca²^+^ via carboxyl/hydroxyl groups at a low pH; this displaces phosphates from their soil adsorption sites to enhance solubility (58). While the profiles of organic acids vary among PSMs, this study observed the metabolic enrichment of key acids, such as malic, ketoglutaric, and citric acid among others, thus supporting the dissolution of phosphate.

HT-PGPR accumulate organic osmolytes, such as proline, betaine, and other amino acids, in response to various abiotic stresses, which is more common in saline soils (59). While exposed to salt stress, HT-PGPR may temporarily increase their cytoplasmic contents of K^+^, but the accumulation of osmolytes is a more sustained stress response to prevent water loss (60). The capacity to accumulate proline has been known to correlate with stress tolerance for many years. Proline is considered to act as an osmolyte, an ROS scavenger, and a molecular chaperone that stabilizes the structure of proteins, thereby protecting the cells from the damage caused by stress (61). Proline accumulates in many species of plants in response to different environmental stresses, including drought, high salinity, and heavy metals (62). We found proline to be particularly enriched in the supernatant on day 7 of culture. Additionally, organic acids have also been shown to buffer environmental conditions, such as nutrient toxicities or deficiencies, particularly in environments with a high pH, such as saline soil. HT-PGPR release organic acids in unfavorable environmental conditions to act as chelators to increase the availability of nutrients. Malic acid, citric acid, and succinic acid, which are the main organic acids in this system, began to be enriched on day 1 and reached their highest levels on day 7.

It is notable that HT-PGPB serves the plant in saline soil by producing the phytohormone auxin. More than 80% of the rhizosphere bacteria are estimated to produce IAA, a dominant form of auxin that promotes plant growth (63). The primary biosynthetic pathway to IAA is through the metabolism of tryptophan, which can be mediated by plants or soil microorganisms. We found that IAA and tryptophan accumulated in the medium. In addition, seed germination experiments showed that *S. succinus* NG-9 can promote plant growth (Fig. 6). Thus, the strain may have increased the production of tryptophan, thereby promoting the growth of plants through the biosynthesis of auxin.

### Analysis of the activities of phosphatase and phytase

The other way to increase the bioavailability of phosphorus in the soil is related to the release of extracellular enzymes. Phytate is the most abundant form of organic phosphorus in the soil. Phytase transforms phytate into soluble phosphorus that is available to the roots (64). The production of phytase by bacterial isolates could promote the growth of plants in agricultural soils that are deficient in phosphorus (65). The quantification of the amount of phytase produced by this isolate in the NBRIP liquid medium amended with the different concentrations of phosphate showed that the activity of phytase was 2.48 ± 0.07 and 1.91 ± 0.05 U/mL up to 7 days of incubation, which indicates that *S. succinus* NG-9 clearly possesses phytase activity (Fig. S4A and B). This study showed a higher activity of phytase than that obtained by Belkebla et al. (66), which was 0.168 ± 0.002 U/mL. Therefore, this strain may be considered to be highly likely to produce active phytase. Similarly, *S. succinus* NG-9 biosynthesizes acid and alkaline phosphatases, which are enzymes involved in the process of phosphate mineralization that affects plant growth. Both alkaline phosphatase and acid phosphatase were secreted by *S. succinus* NG-9 during growth on NBRIP that contained tricalcium phosphate or calcium phytate. The maximum activity of alkaline phosphatase was produced by *S. succinus* NG-9 (0.35 ± 0.02 U/mL) on day 7 of incubation in the medium with calcium phytate. The maximum activity of acid phosphatase was demonstrated by *S. succinus* NG-9 (0.33 ± 0.02 U/mL) on day 7 of incubation on the medium that contained calcium phytate.

### Effects of inoculation with *S. succinus* NG-9 on seed germination under saline-alkali stress

**Fig 5:**
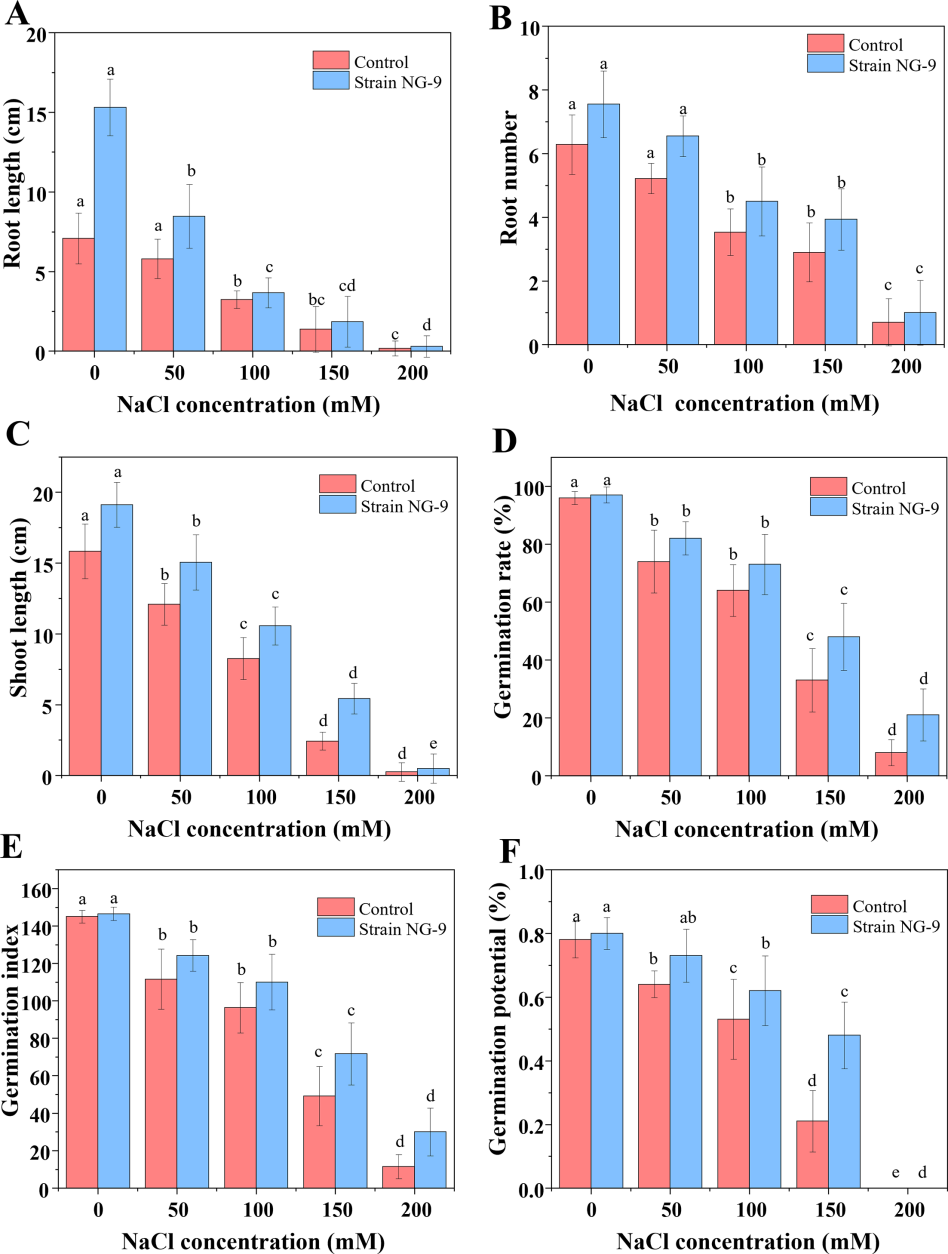
Specific effects of inoculation with *S. succinus* NG-9 on root length (**A**), root number (**B**), shoot length (**C**), germination rate (**D**), germination index (**E**), and germination potential (**F**) of wheat seeds. Different lowercase letters indicate the significant differences (*P* < 0.05).

We performed seed germination tests to examine the stimulatory effects of *S. succinus* NG-9 on the growth of wheat seeds under saline-alkali stress. *As shown in* Figure 5 and Fig. S5 the ability of *S. succinus* NG-9 to promote plant growth was demonstrated by its significant enhancement of wheat germination and early seedling development under saline-alkali stress. Specifically, inoculation with *S. succinus* NG-9 increased the relative germination rate, vigor index, germination index, and germination potential compared to untreated controls. These results suggest that strain NG-9 mitigates the inhibitory effects of saline-alkali stress, potentially through mechanisms such as nutrient solubilization, phytohormone production, or osmotic regulation. The ability of NG-9 to enhance the germination of the seeds was limited under non-saline conditions. However, a higher level of salinity caused a more obvious effect of NG-9 on the wheat seeds compared with the uninoculated salt-stressed seeds. Moreover, the relative germination of the seeds increased significantly in parallel with the levels of NaCl. When treated with NG-9, a concentration of 200 mM provided the best relative germination, with a 159.90% increase over the non-saline conditions. In addition, under the treatment with 200 mM NaCl, the inoculation with NG-9 enhanced the germination index and vigor index by 162.5% and 333.3%, respectively, compared with their uninoculated counterparts. In contrast, under non-stress conditions, the germination index and vitality index when the seedlings were inoculated with strain NG-9 only increased by 1.1% and 43.0%, respectively, compared with that of the seeds not inoculated with NG-9. The inoculation of strain NG-9 increased the germination of wheat seeds in either saline or non-saline conditions, and 150 mM NaCl induced the most rapid seed germination on day 3. These results indicated that NG-9 could promote the germination of wheat seeds under salt stress. Moreover, NG-9 was more effective at promoting the growth of plants under higher salt stress conditions (150 and 200  mM NaCl).

**Fig 6:**
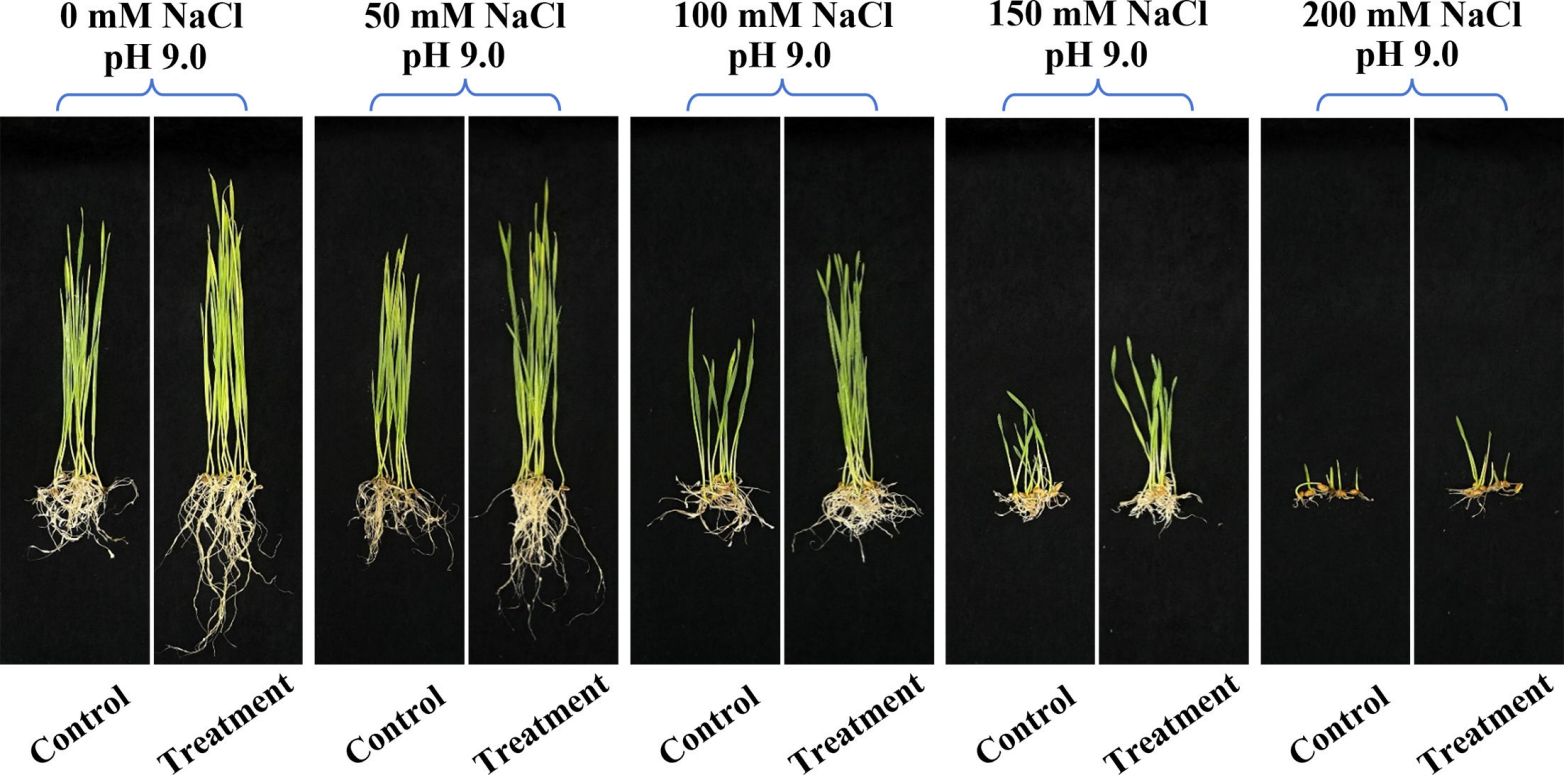
Overall observation on the effects of inoculation with *S. succinus* NG-9 on the growth of wheat seedings at simulated NaCl concentrations and pH 9.0. C, control; T, treatment.

The results indicated that the inoculation with strain NG-9 greatly improved the wheat growth under a range of salt stress conditions (Fig. 6). The results in Fig. 5 show that the salt stress significantly reduced the root length and number, shoot length, and germination rate. However, the application of the strain significantly alleviated this reduction compared with the uninoculated salt-stressed samples. When the seeds were exposed to varying levels of salt stress, their germination rate when inoculated with this strain increased by 1% (0 mM NaCl), 10.8% (50 mM NaCl), 14.1% (100 mM NaCl), 45.5% (150 mM NaCl), and 162.5% (200 mM NaCl) compared to the uninoculated seeds. The roots of the wheat seedlings grew longer following the inoculation of strain NG-9 under both saline and non-saline conditions. The results indicated that the lack of salt stress had the greatest effect on root length with a 2.2-fold increase compared to the uninoculated seeds. It also increased the root length by 46.0% under 50 mM NaCl, 13.3% under 100 mM NaCl, 34.1% under 150 mM NaCl, and 66.7% under 200 mM NaCl. Under the treatment with 200 mM NaCl, inoculation with NG-9 enhanced the root number and shoot length by 28.6% and 59.2%, respectively, compared with their uninoculated counterparts.

## DISCUSSION

*Staphylococcus succinus* NG-9 isolated from saline-alkali soil demonstrated exceptional abilities to solubilize phosphate and dissolved 450.36 mg/L of inorganic phosphorus and 333.15 mg/L of organic phosphorus. While previous studies reported that the strains associated with saline conditions could tolerate up to 1,600 mM NaCl (9.4% wt/vol) and solubilize phosphorus at 320 mM NaCl (1.9% wt/vol) (67), few could maintain activity at 13.0% NaCl, except for certain halophilic bacteria (68). NG-9 not only functions across 1.0%–13.0% NaCl but peaks at 5.0% NaCl, thus surpassing conventional phosphate-solubilizing microorganisms. This exceptional tolerance probably stems from its genomic arsenal, including the osmoprotectant biosynthetic genes (*betA*, *betB*, *proC*) and robust ion homeostasis systems (*trkH*, *mrp*), which act synergistically to mitigate the toxicity of ions while maintaining essential metabolic functions.

Strain NG-9 employs a multifaceted strategy to solubilize phosphate that is particularly effective under saline-alkali conditions. The ability of this strain to acidify its environment through the release of protons during the assimilation of NH_4_^+^, which entails the conversion of NH_4_^+^ to NH_3_, complements (69) its secretion of organic acids, including gluconate via *gdh* activity, to solubilize insoluble phosphates. This dual mechanism is particularly valuable in saline-alkali soils with a high pH where the availability of phosphorus is typically limited. The preference of this strain for glucose as a C source and NH_4_Cl as an N source is consistent with the findings of Li et al. (70) and confirms that these substrates optimally support the solubilization of phosphate in PSMs. Furthermore, the production of siderophores (36.63 ± 0.06%) by strain NG-9 enhances the acquisition of iron under alkaline conditions where the bioavailability of phosphorus is restricted (71), while its ACC deaminase activity (7.37 ± 0.40 µmol α-ketobutyric acid/mg protein/h) helps to mitigate the stress responses mediated by ethylene in plants.

The ability of strain NG-9 to promote plant growth under saline stress is mediated through an integrated system of IAA production, EPS secretion, and biofilm formation. The production of phytohormones, such as IAA, is one of the most important features exhibited by PGPR in the rhizosphere. Under saline-alkali stress, IAA is needed to enhance the lengths of roots and their surface areas to ensure the availability of nutrients and balance the influx of ions (72). IAA helps the seeds to germinate and form longer roots with more root hairs, which indirectly helps the plant to take up more nutrients. It has been reported that 80% of the rhizobacterial populations release IAA, which is a physiologically active auxin. Babar et al. (73) reported that the IAA produced by halotolerant strains of *Alcaligenes faecalis* enhanced the overall performance of plants under salt stress. Even at greater levels of NaCl, the halotolerant NG-9 strain secreted IAA, which is an unusual and beneficial characteristic of halotolerant microbes because such halotolerant strains are more likely to endure the biosynthesis of IAA and enable the plants to access this important growth-promoting phytohormone even in a saline environment. Additionally, the production of EPS (1.04 g/L) and formation of biofilm (peaking at an OD_590_ of 0.76 under 5.0% NaCl) by strain NG-9 mirror the stress-mitigation strategies observed in *Bacillus cereus* KTES (74) and other salt-resistant PGPR (75). The biofilm dynamics, which increase at a moderate salinity before declining under extreme stress, reflect a balance between protective aggregation and metabolic sustainability; this is consistent with the microbial responses along stress gradients (76). These interconnected mechanisms position NG-9 as a comprehensive bioinoculant to improve the performance of crops in saline-alkali environments.

**Fig 7:**
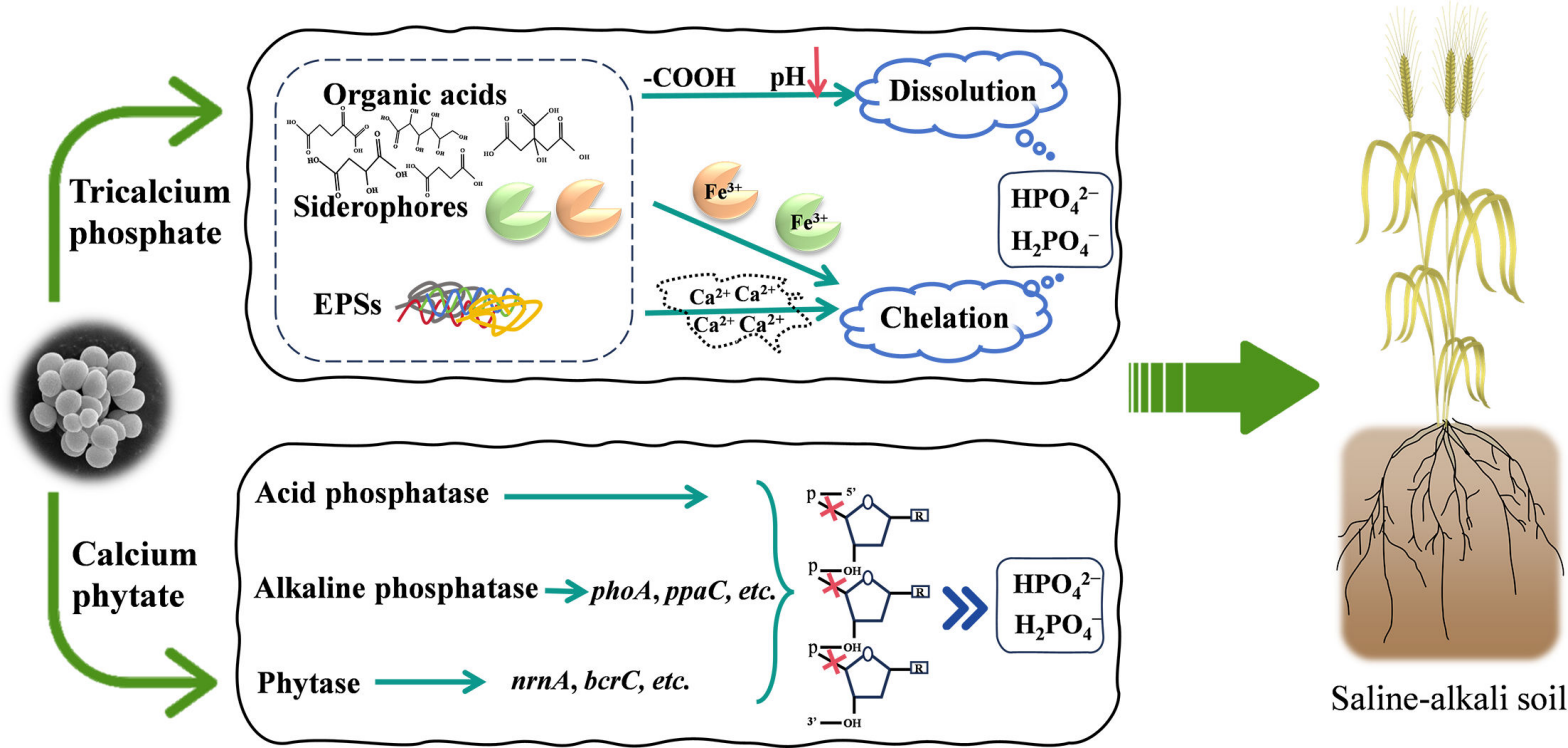
Underlying mechanisms for organic/inorganic phosphate solubilization by *S. succinus* NG-9 under saline-alkali conditions.

Basically, the plants grown under saline-alkali stress suffer from osmotic stress and nutritional imbalances. The phosphorus-solubilizing *S. succinus* NG-9 that is tolerant to saline-alkali conditions can increase the content of soluble phosphate, thereby increasing the absorption of nutrients by plants under saline-alkali stress. This may be one of the reasons why the plants inoculated with *S. succinus* NG-9 grew better. In addition to its activity of ACC deaminase and production of EPS, *S. succinus* NG-9 can produce the growth hormone IAA, which may stimulate the growth of wheat. Based on these data from quantitative, genomic, metabolic, and enzymatic analyses, the underlying mechanisms of *S. succinus* NG-9 to enhance the germination of wheat seeds and seedling growth under saline-alkali conditions can be hypothesized as shown in Fig. 7.

### Conclusions

A highly efficient organic/inorganic phosphate-solubilizing strain was isolated from a saline-alkali soil sample and identified as *Staphylococcus succinus* NG-9. This strain is highly tolerant to high levels of salt-alkali and adaptable to different environments. It was further confirmed to possess multifaceted plant growth-promoting traits under saline-alkali stress, including the solubilization of organic/inorganic phosphate, production of siderophores, secretion of IAA, ACC deaminase activity, and the formation of biofilm, which contribute to an improvement in plant growth. The genomic data annotated several key genes associated with phosphate solubilization, saline-alkali tolerance, biofilm formation, and plant growth regulation. Untargeted metabolomic data and enzyme assays supported the hypothesis that the production of organic acids and osmotolerants and phytase are the predominant mechanisms that enable this strain to solubilize phosphorus under saline-alkali stress. More significantly, inoculation with *S. succinus* NG-9 significantly improved the germination of seeds and the growth of wheat seedlings under simulated saline-alkali conditions. Taken together, these findings will provide a theoretical basis for the development of microbial fertilizers using *S. succinus* that are suitable for application in saline-alkaline lands.

## Data Availability

Data will be made available on request.

## SUPPLEMENTAL MATERIAL

Tables S1 and S2, and Figures S1 to S5 spectrum.00490-25-s0001.pdf Table S1: Phenotypic characteristics of *S. succinus* NG-9. Table S2: Plant growth-promoting traits of *S. succinus* NG-9 under saline-alkaline conditions. Fig. S1: pH changes in culture media during phosphate solubilization by *S. succinus* NG-9 under different conditions. Fig. S2: Effects of different carbon (A and B) and nitrogen sources (C and D) on phosphate solubilization and final pH values of fermented broth. Fig. S3: Effects of different NaCl concentrations on biofilm formation by *S. succinus* NG-9. Fig. S4: Activities of acid phosphatase, alkaline phosphatase and phytase when *S. succinus* NG-9 was exposed to inorganic phosphate (A) and organic phosphate (B), respectively. Fig. S5: Specific effects of inoculation with *S. succinus* NG-9 on relative germination (A), vigor index (B) of wheat seeds.

ASM does not own the copyrights to Supplemental Material that may be linked to, or accessed through, an article. The authors have granted ASM a non-exclusive, world-wide license to publish the Supplemental Material files. Please contact the corresponding author directly for reuse.
