# Supplementary material for: Simultaneous organic/inorganic phosphate solubilization by Staphylococcus succinus NG-9 under saline-alkali conditions: Insights into its characteristics, mechanisms, and potential applications
Source: Microbiol Spectr. 2025 Jul 31;13(9):e00490-25. doi: 10.1128/spectrum.00490-25 (PMC12403767; doi:10.1128/spectrum.00490-25)
Supplement: Tables S1 and S2, and Figures S1 to S5 — Table S1: Phenotypic characteristics of S. succinus NG-9. Table S2: Plant growth-promoting traits of S. succinus NG-9 under saline-alkaline conditions. Fig. S1: pH changes in culture media during phosphate solubilization by S. succinus NG-9 under different conditions. Fig. S2: Effects of different carbon (A and B) and nitrogen sources (C and D) on phosphate solubilization and final pH values of fermented broth. Fig. S3: Effects of different NaCl concentrations on biofilm formation by S. succinus NG-9. Fig. S4: Activities of acid phosphatase, alkaline phosphatase and phytase when S. succinus NG-9 was exposed to inorganic phosphate (A) and organic phosphate (B), respectively. Fig. S5: Specific effects of inoculation with S. succinus NG-9 on relative germination (A), vigor index (B) of wheat seeds. [file spectrum.00490-25-s0001.pdf]

Microbiology Spectrum

Supplementary Material for

Simultaneous organic/inorganic phosphate solubilization by *Staphylococcus succinus* NG-9 under saline-alkali conditions: Insights into its characteristics, mechanisms, and potential applications

Xue Xie[a†], Zhongshun Xu[a†], Longzhan Gan[a*], Chunbo Dong[a], Ting Zhang[a], Ya Huang[a], Tengxia He[a], Yongqiang Tian[b], Xiao Zou[a*]

[a]*Institute of Fungus Resources, Guizhou Key Laboratory of Agricultural Microbiology/Key Laboratory of Plant Resource Conservation and Germplasm Innovation in Mountainous Region (Ministry of Education), College of Life Sciences, Guizhou University, Guiyang 550025, Guizhou Province, China*

[b]*Key Laboratory of Leather Chemistry and Engineering (Ministry of Education), College of Biomass Science and Engineering, Sichuan University, Chengdu 610065, Sichuan Province, China*

†These authors contributed equally to this work

***Correspondence:** Longzhan Gan E-mail: lzgan@gzu.edu.cn

Xiao Zou E-mail: xzou@gzu.edu.cn

Table S1. Phenotypic characteristics of *S. succinus* NG-9

Characteristic	Test result
Urease activity	+
Indole production	+
Oxidase activity	–
Catalase activity	–
Voges–Proskauer test	–

Note: +, Positive; –, Negative.

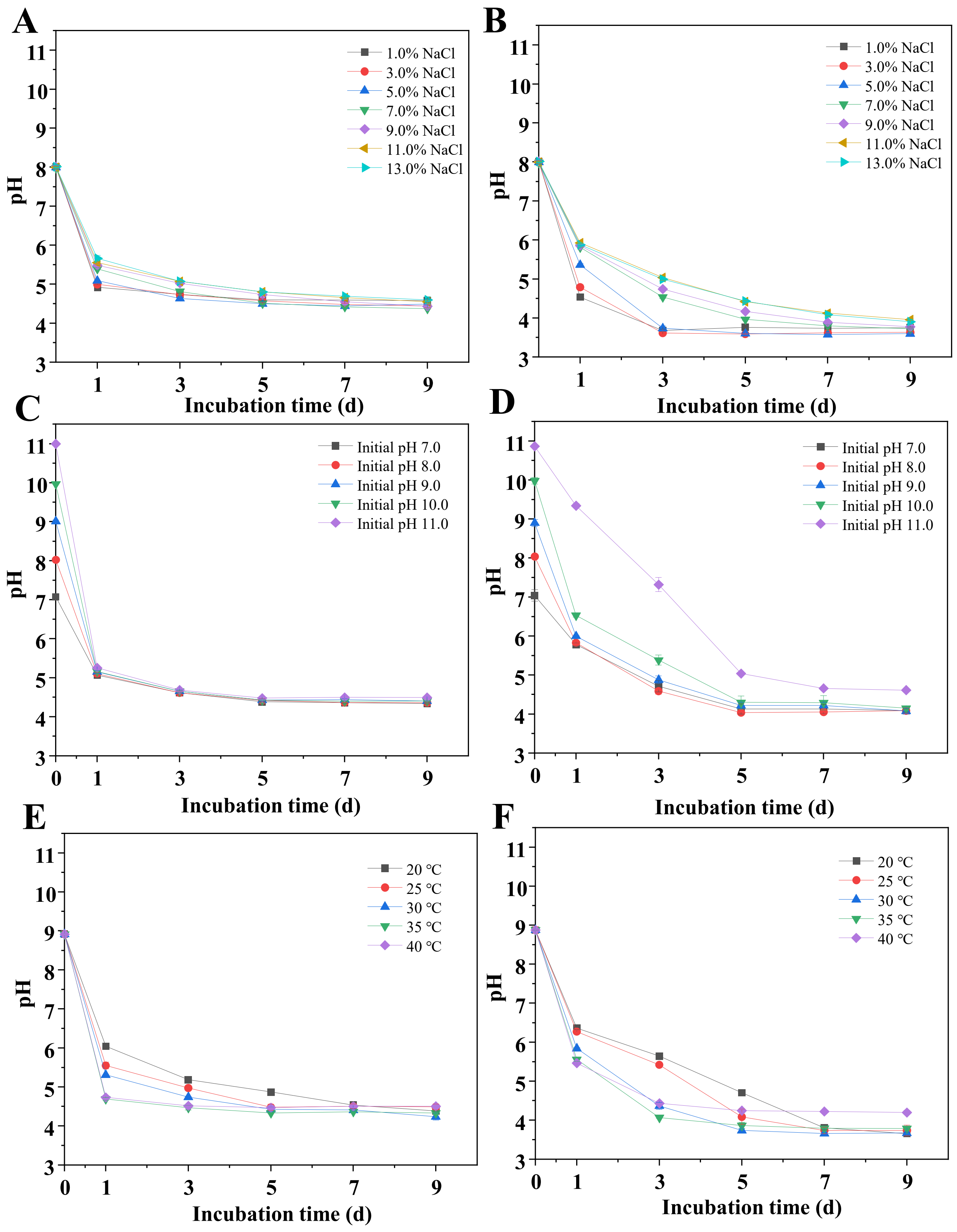

Fig. S1. pH changes in culture media during phosphate solubilization by *S. succinus* NG-9 under different conditions. NaCl concentrations (A and B for inorganic and organic phosphates, respectively); initial pH values (C and D for inorganic and organic phosphates, respectively); culture temperatures (E and F for inorganic and organic phosphates, respectively).

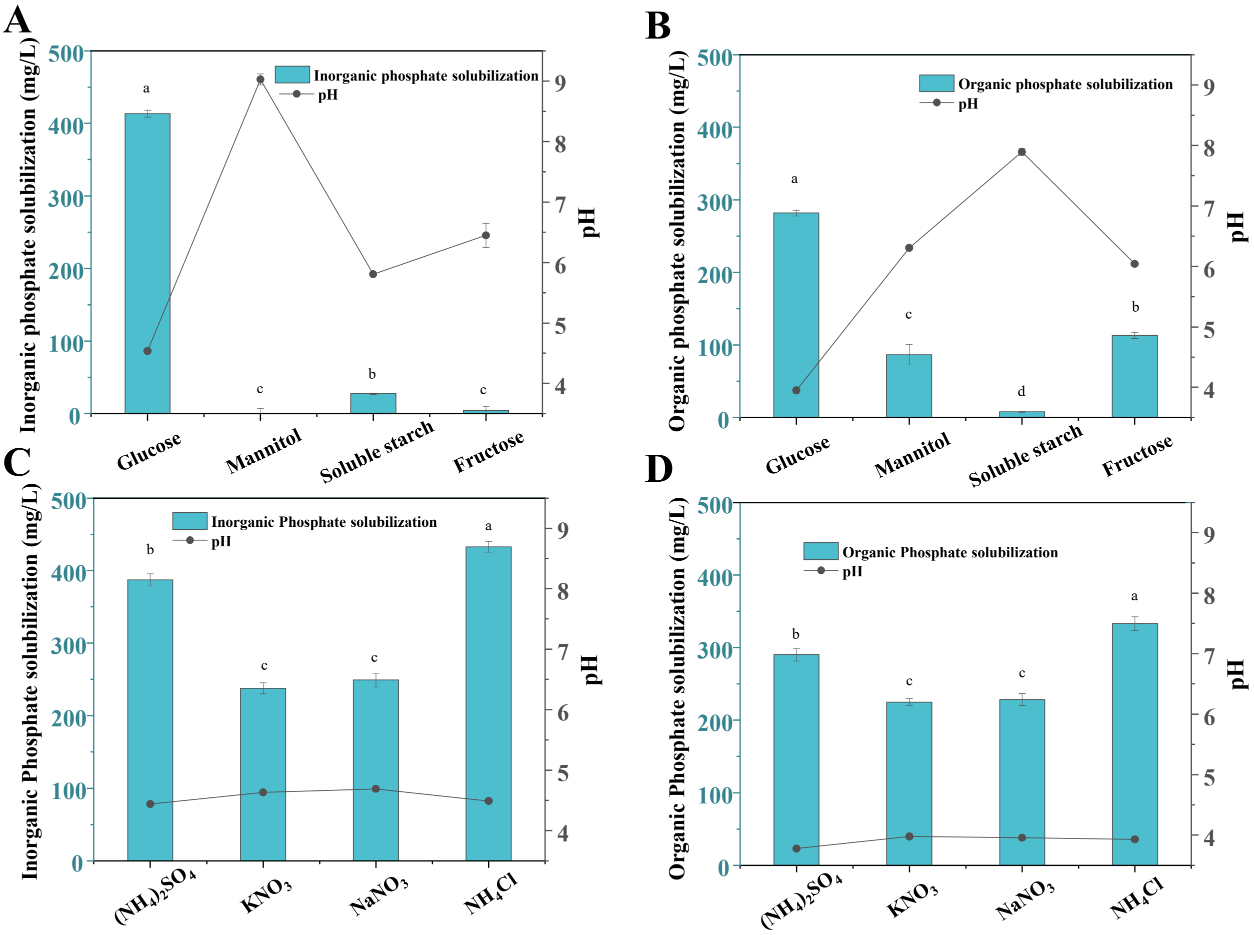

Fig. S2. Effects of different carbon (A and B) and nitrogen sources (C and D) on phosphate solubilization and final pH values of fermented broth. Different lowercase letters indicate the significant differences ($p < 0.05$).

Table S2. Plant growth-promoting traits of *S. succinus* NG-9 under saline-alkaline conditions

**Characteristic**	**Yield/Activity**
EPS production (g/L)	1.04±0.33
Siderophore production (%)	36.63±0.06
IAA production (μg/ml)	4.14 ± 0.69
ACC deaminase activity (μmol α-ketobutyric acid/mg protein/h)	7.37±0.40

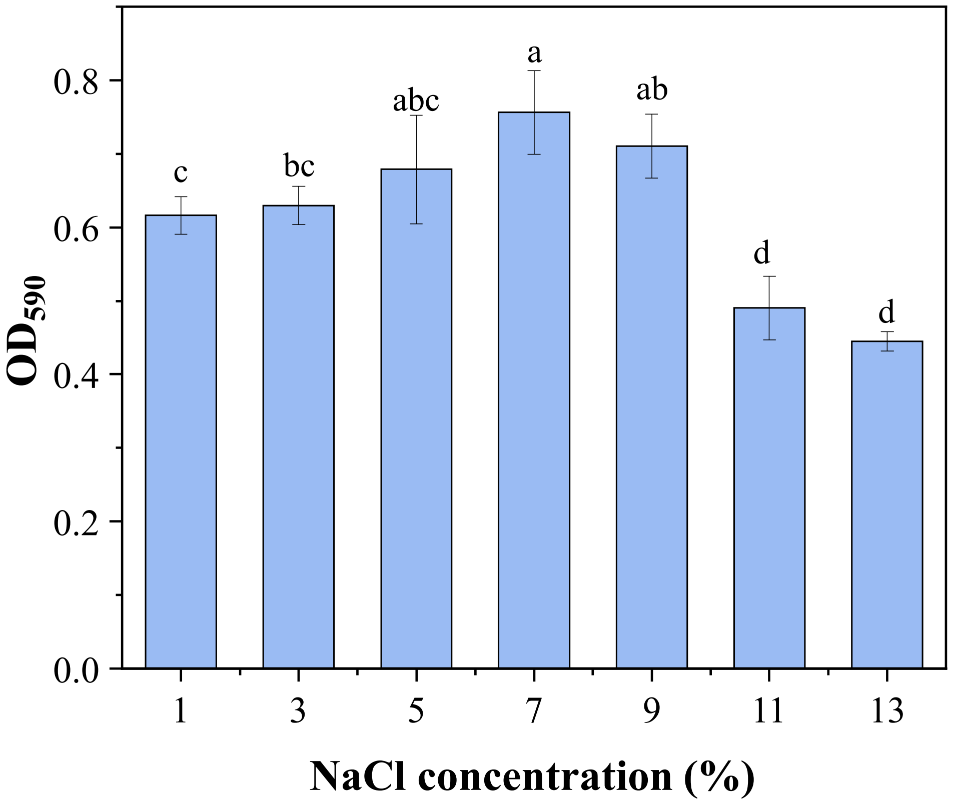

Fig. S3. Effects of different NaCl concentrations on biofilm formation by *S. succinus* NG-9. Different lowercase letters indicate the significant differences ($p < 0.05$).

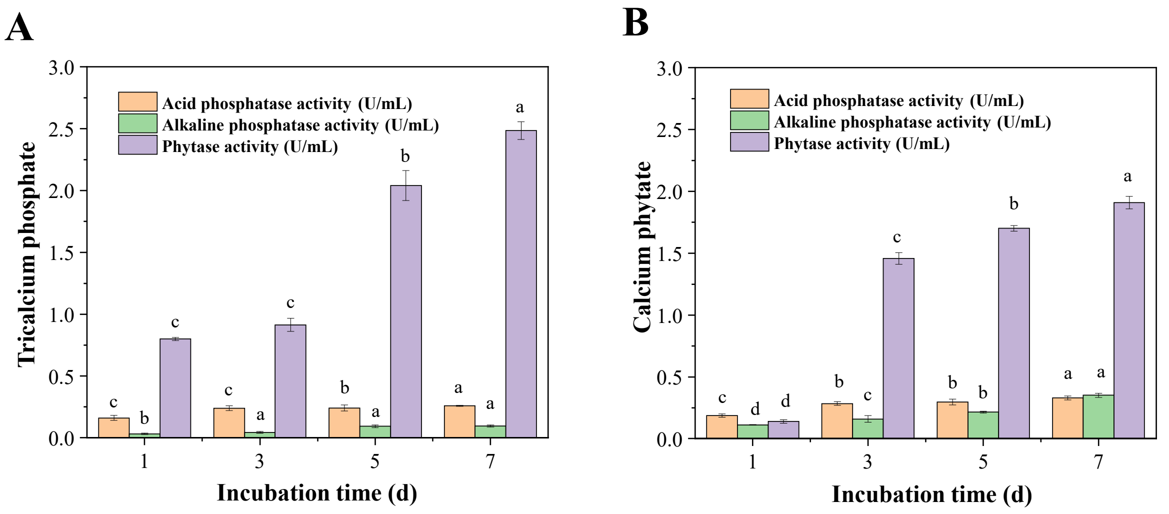

Fig. S4. Activities of acid phosphatase, alkaline phosphatase and phytase when *S. succinus* NG-9 was exposed to inorganic phosphate (A) and organic phosphate (B), respectively. Different lowercase letters indicate the significant differences ($p < 0.05$).

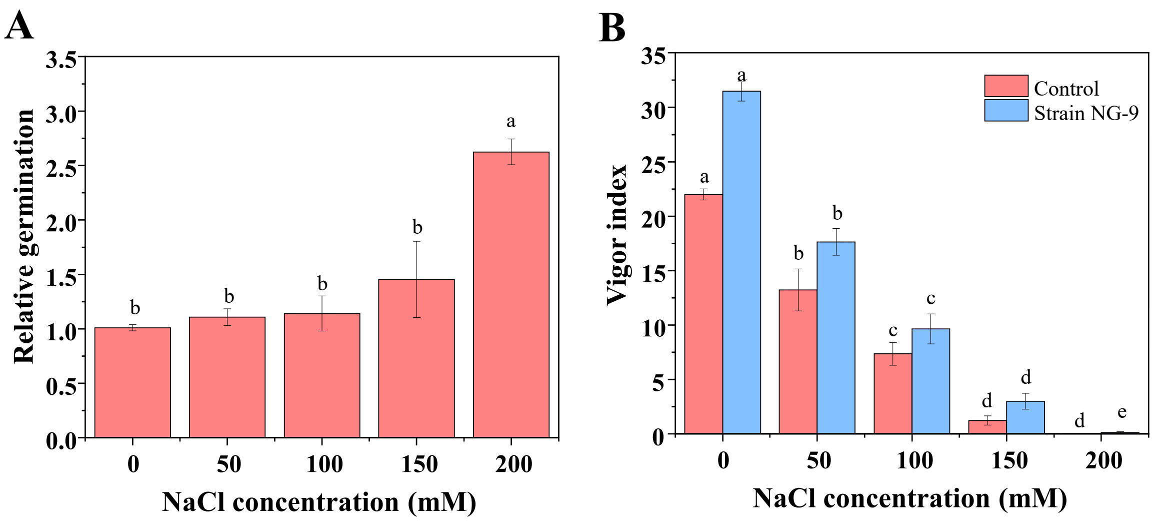

Fig. S5. Specific effects of inoculation with *S. succinus* NG-9 on relative germination (A), vigor index (B) of wheat seeds. Different lowercase letters indicate the significant differences ($p < 0.05$).
